# A Rare Case of Postmenopausal Hirsutism Associated With a Serous Cystadenofibroma of the Ovary

**DOI:** 10.7759/cureus.63077

**Published:** 2024-06-24

**Authors:** Grishma Pokharel, Shreya Bhandari, Rachel Bier, Stephanie Rosales

**Affiliations:** 1 Internal Medicine, Englewood Hospital and Medical Center, Englewood, USA; 2 Endocrinology, Diabetes and Metabolism, Endocrinology Consultants of Englewood, Englewood, USA

**Keywords:** serous cystadenofibroma, hirsutism, androgen access, benign epithelial tumors of ovary, hirsutism and ovarian tumors, post menopausal hirsutism

## Abstract

Hirsutism in females is most commonly associated with polycystic ovarian syndrome, but can also result from congenital adrenal hyperplasia and ovarian tumors like granulosa cell tumors, Sertoli-Leydig cell tumors, and hilus cell tumors. We present a case of a 54-year-old female with hirsutism, diagnosed with ovarian cystadenofibroma. She had a history of premature ovarian failure at the age of 35 and presented with new onset chin and upper lip hair, and scalp hair loss. Labs showed elevated total testosterone, normal dehydroepiandrosterone (DHEA) and sex hormone-binding globulin (SHBG), low estradiol, and postmenopausal range anti-Mullerian hormone (AMH), follicle-stimulating hormone (FSH), luteinizing hormone (LH), and prolactin. Cytogenetic testing showed a normal XX karyotype. Initial transvaginal ultrasound revealed a thickened endometrial stripe and unremarkable ovaries. Repeat ultrasound and MRI noted persistent endometrial thickening and a solid-cystic structure in the left ovary. The patient underwent total hysterectomy, bilateral salpingo-oophorectomy, and sentinel lymph node dissection. Endometrial biopsy showed FIGO grade 1 endometrioid carcinoma, and the left ovary biopsy revealed benign serous cystadenofibroma and endometriosis. Postoperatively, hirsutism resolved and testosterone levels normalized. Hirsutism in postmenopausal women should prompt evaluation for adrenal or ovarian sources, including tumors. Ovarian tumors cause about 1% of hirsutism cases. Our case highlights the need for thorough evaluation, as benign ovarian tumors can also cause androgen excess and associated conditions like endometrial cancer.

## Introduction

Hirsutism is defined as the excessive growth of hair in androgen-dependent parts of the body. Irrespective of the cause, hirsutism can cause significant emotional stress and mental anguish [1]. In females, hirsutism is most commonly associated with polycystic ovarian syndrome. Other causes include congenital adrenal hyperplasia, idiopathic hyperandrogenemia, idiopathic hirsutism, Cushing syndrome, hyperprolactinemia, and ovarian tumors. The classically seen tumors are granulosa cell tumor, Sertoli-Leydig cell tumor, and hilus cell tumor [2,3]. We present a case of a 54-year-old female who presented with hirsutism and was diagnosed with ovarian cystadenofibroma.

## Case presentation

A 54-year-old female G0P0SAB1 with a history of premature ovarian failure since age 35, presented with complaints of new onset chin and upper lip hair (Ferriman-Gallwey score=12), and loss of scalp hair for one year. Labs were significant for elevated total testosterone, low estradiol, and progesterone as illustrated in Table 1.

**Table 1:** Laboratory findings FSH: Follicle-stimulating hormone; LH: Luteinizing hormone; SHBG: Sex hormone-binding globulin; DHEA: Dehydroepiandrosterone; TSH: Thyroid-stimulating hormone; TSI: Thyroid-stimulating immunoglobulin; AM: Ante Meridiem.

Test name	Lab value	Reference range (Postmenopausal)
FSH	54.4 mIU/ml	26.72-133.41 mIU/ml
LH	24.30 mIU/ml	5.16-61.99 mIU/ml
Estradiol	19.2 pg/ml	22.4-517.0
Prolactin	12.4 ng/ml	5.2-26.5 ng/ml
Testosterone total	63.7 ng/dl	12.4-35.8 ng/dl
Testosterone free	1.7 pg/ml	0.3-4.4 pg/ml
Androstenedione	0.4 ng/ml	0.130-0.820 ng/ml
Progesterone	0.21 ng/ml	0.33-24.0 ng/ml
17-hydroxyprogesterone	18.24 ng/dl	<206 ng/dl
SHBG	18.5 nmol/l	11.7-137.2 nmol/l
DHEAs	85.2 ug/dl	56.2-282.9 ug/dl
Insulin-like growth factor I	127 ng/ml	17-235 ng/ml
TSH	3.783 uIU/ml	0.350-4.940 uIU/ml
Free T4	0.90 ng/dl	0.70-1.48 ng/dl
Free T3	2.7 pg/ml	1.6-3.9 pg/ml
T3 total	119 ng/dl	87-178 ng/ml
TRAb	<1.10 IU/L	<1.75 IU/L
TSI	<0.1 IU/L	0-0.55 IU/L
Total AM cortisol	12.7 ug/dl	3.7-19.4 ug/dl

DHEA and SHBG were within normal limits. AMH, FSH, LH, and prolactin were within the postmenopausal range. Cytogenetic testing was ordered and revealed a normal XX karyotype with a GTG banding pattern observed in all cells. Transvaginal ultrasound revealed a thickened and echogenic endometrial stripe, with unremarkable ovaries for which close observation was recommended. Given the high suspicion of an ovarian tumor, we recommended a repeat ultrasound and MRI of the pelvis. The transvaginal ultrasound revealed persistent endometrial thickening, and a heterogenous solid and cystic structure in the left ovary. The pelvic MRI noted an endometrial mass of 6.4 x 2.2 x 4 cm and a 3 cm ovarian lesion, requiring further characterization, however bilateral adrenal gland was unremarkable. The patient underwent total hysterectomy, b/l salpingo-oophorectomy, and sentinel lymph node dissection. Endometrial biopsy showed FIGO grade 1 endometrioid carcinoma. The biopsy from the left ovary showed benign serous cystadenofibroma and endometriosis. Postoperatively, at three months, the patient had no further hirsutism, and testosterone levels normalized.

## Discussion

Hirsutism caused by ovarian tumors accounts for approximately 1% of all cases of hirsutism [4]. Although menopause is a relatively hyperandrogenic state, the development of hirsutism or virilizing features should never be considered normal. Cystadenomas are relatively benign ovarian epithelial tumors that have rarely been implicated as the cause of hyperandrogenism [5]. Initial workup with imaging did not reveal any ovarian mass in our case, however, the endometrial thickening observed can be hypothesized to be secondary to androgen access from undiscovered mass, peripherally converting to estrogen-causing endometrial cancer. A high clinical suspicion of a testosterone-producing ovarian tumor led to repeat imaging noting an ovarian and incidental endometrial carcinoma. Surgery was curative for both hirsutism and endometrial cancer. As noted in our case report, we detected androgen excess arising from a benign epithelial tumor.

## Conclusions

Hirsutism in females often comes up as a cosmetic or dermatological concern. Beyond just the negative psychological impact on the quality of life of a patient, it can be the first and only manifestation of underlying life-threatening disease. Hirsutism in females should always prompt an extensive evaluation, with a high degree of suspicion for adrenal and ovarian tumors. While malignant ovarian tumors have been highly recognized to be associated, our finding of benign tumor and recovery after the excision highlights the need for further studies in this sector.
